# Incidence, risk factors, clinical and cognitive impact of secondary hemorrhage after spontaneous aneurysmal subarachnoid hemorrhage

**DOI:** 10.1016/j.bas.2026.106073

**Published:** 2026-05-02

**Authors:** Vera Marschal, Andreas Ziebart, Maryam Abdoullahi, Daniel Werkmann, Ralph König, Thomas Kapapa, Benjamin Mayer, Johannes Rosskopf, Lennart Marschal, Christian Rainer Wirtz, Andrej Pala, Gregor Durner

**Affiliations:** a Department of Neurosurgery, University Hospital Ulm, Ulm, Germany; b Department of Neurosurgery, University of Ulm, District Hospital Gunzburg, Gunzburg, Germany; c Institute of Epidemiology and Medical Biometry, University of Ulm, Ulm, Germany; d Department of Neuroradiology, University Hospital Ulm, District Hospital Günzburg, Germany; e Department of Internal Medicine, University Hospital Ulm, Klinikum Heidenheim gGmbH, Germany

**Keywords:** Secondary hemorrhagic complications, Subarachnoid hemorrhage, External ventricular drainage, Antiplatelet therapy, Cognitive outcome, Functional outcome

## Abstract

**Introduction:**

Evidence on secondary hemorrhage (SH) in patients with spontaneous aneurysmal subarachnoid hemorrhage (aSAH) is limited. Most studies focus external ventricular drain (EVD)-related bleeding, while data on hemorrhage types, particularly under peri-interventional antiplatelet therapy (PIAT), are scarce.

**Research question:**

To investigate the incidence, risk factors, and clinical impact of SH after aSAH, including the role of PIAT.

**Material and methods:**

In this registry-based two-centre study we retrospectively analyzed 170 patients with aSAH treated between September 2021 and June 2025. EVD placement was performed in 128 patients; 120 underwent endovascular treatment, and 78 received PIAT. SH was classified as intracranial or peripheral. Functional and cognitive outcome were assessed using the modified Rankin Scale and the Montreal Cognitive Assessment, respectively, at admission, discharge, and three months.

**Results:**

SH occurred in 39% of patients. Intracranial hemorrhages predominated, with drain-associated (29%), aneurysm rebleeding (27%), and access-related hemorrhages (21%) being most frequent. EVD placement was independently associated with SH (OR 5.07, p = 0.002). No significant association between PIAT and SH was observed. SH was associated with worse functional and cognitive outcomes, however, age and WFNS grade were the main independent predictors.

**Discussion and conclusion:**

SH after aSAH is common and reflects a heterogeneous spectrum of bleeding events. The association with EVD likely reflects disease severity rather than causality. No association between PIAT and SH was found, although analyses were limited. SH appears to be a marker of disease severity associated with worse early outcomes, emphasizing careful procedural management and individualized risk assessment.

## Highlights

• Secondary hemorrhage occurred in 39% after aneurysmal SAH.
• Intracranial bleeding dominated, especially drain-associated events.
• EVD placement was strongly associated with secondary hemorrhage.
• PIAT showed no significant association with hemorrhage risk.
• SH worsened early outcome but not long-term outcome independently.

## 1 Introduction

Spontaneous subarachnoid hemorrhage (SAH) is a life-threatening condition with a high rate of morbidity and mortality (Ziebart et al., 2024). Common complications following SAH include rebleeding from the ruptured aneurysm, hydrocephalus, cerebral severe vasospasm (SV), delayed cerebral ischemia (DCI), and infarction (Lindegaard et al., 1988; Suarez-Rivera, 1998; Ohkuma et al., 2001; Tanno et al., 2007; Diringer et al., 2011; Westermaier et al., 2012; Fu et al., 2013). Apart from aneurysmal rebleeding, secondary hemorrhage (SH), such as intraparenchymal or ventricular hemorrhages, may occur due to surgical or interventional procedures and associated therapies.

A significant proportion of patients with aneurysmal SAH undergo endovascular treatment, which can require periinterventional antiplatelet therapy (PIAT) to prevent thrombo-occlusive events, especially when using stents, flow diverters, or intrasaccular devices such as the Woven Endo Bridge (WEB) (Wang et al., 2009; Fischer et al., 2012; Zhao et al., 2018). However, PIAT carries an inherent risk of bleeding, which is particularly concerning in the acute phase of SAH when invasive procedures such as external ventricular drain (EVD) placement or decompressive craniectomy may be required.

EVD placement is frequently performed early after SAH to manage hydrocephalus and monitor intracranial pressure. While generally safe, EVD insertion carries risks including infection, malposition, and hemorrhagic complications. The use of antiplatelet therapy in proximity to EVD placement has been associated with an increased risk of puncture channel bleeding (PCB). Several studies demonstrated a correlation between PIAT and PCB (Kung et al., 2011; Sims-Williams et al., 2014; Bruder et al., 2015; Woo et al., 2019). In contrast, other studies, such as that by Maher Hulou et al. (2022), found no association between antiplatelet therapy and EVD-related bleeding, highlighting the need for further investigation (Maher Hulou et al., 2022). However, existing studies have primarily focused on specific subtypes of SH, particularly EVD-related bleeding (Darkwah Oppong et al., 2020; Lee et al., 2023), while comprehensive analyses integrating different types of intracranial hemorrhages (IH) and peripheral hemorrhages (PH), the role of PIAT, and their impact on both functional and cognitive outcomes remain limited.

Therefore, the aim of this study was to provide a comprehensive assessment of SH after aSAH. The primary objective was to evaluate the incidence and risk factors of SH. Secondary objectives included (1) the characterization of different hemorrhage types and their origins, (2) the analysis of the association between PIAT and SH, and (3) the assessment of functional and cognitive outcomes using the modified Rankin Scale (mRS) and the Montreal Cognitive Assessment (MoCA). We hypothesized that SH is associated with worse clinical and cognitive outcomes and that both procedural and pharmacological factors contribute to its occurrence.

## 2 Materials and methods

### 2.1 Study design

Data were collected from a prospectively maintained registry including all patients with spontaneous aneurysmal SAH (aSAH) treated at two centers (District hospital Günzburg and University Hospital Ulm) between 09/2021 and 06/2025. Out of 218 patients with spontaneous SAH we analyzed data from 170 patient with aSAH. The study was approved by the local ethics committee (Approval No. 280/21) and conducted in accordance with the principles outlined in the Declaration of Helsinki. Informed consent for the use of anonymized medical data for research purposes was obtained from all patients or their legally authorized representatives. Inclusion criteria consisted of: aneurysmal SAH, patient age≥18 years, signed informed consent form (by patient or legal representative). Exclusion criteria were therefore: non-aneurysmal SAH, patient age <18 years and non-signed informed consent form.

### 2.2 Participant characteristics and clinical assessment

Upon admission, various clinical and demographic parameters were systematically documented. Demographic data included patient age and sex. Diagnostic imaging was assessed through cranial computed tomography (CT), cranial magnetic resonance imaging (cMRI), and digital subtraction angiography (DSA), with particular attention to SH.

Neurological status was evaluated using the World Federation of Neurological Surgeons scale (WFNS) and the Hunt and Hess grading system. For analysis, patients were categorized into two groups based on SAH severity: grades I to III were classified as mild SAH, while grades IV and V were considered severe SAH (Hunt and Hess, 1968; “Report of World Federation of Neurological Surgeons Committee”, 1988).

The chosen treatment modality of the aneurysm was recorded. The PIAT regimen associated with the intervention and the occurrence of intraarterial (i.a.) nimodipine was also documented. In addition, the occurrence of DCI and SV as classified by a board-certified neuroradiologist were recorded as a distinct subgroup. SV was defined as increased blood flow velocity in transcranial doppler ultrasound (TCD) of the basal arterial vessels of more than 120 cm/s, increase of blood flow velocity of more than 30% a day, 30 cm/s/day, or vessel narrowing in CT or DSA. DCI was defined by any new focal neurological impairment, decrease of at least 2 points in the Glasgow coma scale lasting for >1 h and/or a new cerebral infarction on CT or MRI not attributable to treatment or other secondary complications. Notably, when new cerebral infarction was diagnosed with CT or MRI, other sources of infarction such as previous angiography or surgical complications were excluded before diagnosing DCI as the cause. In case of doubt, the event and all interrelated diagnostic measures were excluded from further analysis. Scans taken within 48 h after aneurysm treatment that revealed new cerebral infarction were excluded.

### 2.3 Standard operating procedures for subarachnoid hemorrhages admissions

Time zero was defined as hospital admission and first diagnostic imaging confirming aneurysmal subarachnoid hemorrhage, which was made using CT, cMRI, and/or lumbar puncture. ASAH was suspected in cases of hemorrhage within the basal cisterns, cerebral parenchyma, or subarachnoid regions adjacent to major arteries showing typical bleeding patterns. The bleeding source was identified using CT angiography or DSA. Upon admission, baseline clinical assessment including WFNS, Hunt & Hess grading, and MoCA (in non-intubated patients) was performed.

Acute hydrocephalus was diagnosed based on imaging evidence of ventricular enlargement, narrowed apical sulci in the absence of brain edema, and signs of cerebrospinal fluid diapedesis. In such cases, an EVD or in some cases a lumbar drain was placed as early as possible after detection. A control CT scan was routinely performed within 24 h after EVD placement to confirm catheter position and detect possible procedure-related hemorrhage.

The choice of treatment modality (surgical vs. endovascular) was determined by a multidisciplinary neurovascular team, consisting of at least one experienced neuroradiologist and one experienced vascular neurosurgeon. Aneurysm treatment was performed immediately after stabilization and EVD placement, either on the day of admission (if feasible before 16:00) or on the following day as the first available case.

In patients undergoing endovascular treatment requiring stent-assisted techniques or flow diverters, PIAT was initiated within 24 h prior to or at the start of the procedure according to institutional protocol. Immediate post-procedural CT imaging was routinely performed after aneurysm securing to assess treatment success and detect early SH, including EVD-related or procedure-related bleeding. Postprocedural management was carried out in the intensive care unit (ICU), with continuous input from the neurovascular team throughout the patient's hospital stay. To prevent thromboembolic complications such as deep vein thrombosis or pulmonary embolism, all patients received prophylactic low-molecular-weight heparin starting 24 h after aneurysm treatment. Follow-up (FU) CT imaging was performed in case of neurological deterioration or routinely at least once per week during ICU stay. SV and DCI were assessed during the ICU course using clinical examination, TCD, CTA, or DSA. Functional outcome was assessed using mRS at discharge and at 3-month follow-up. Cognitive outcome (MoCA) was assessed at admission (when feasible) and at 3 months.

### 2.4 Standard operating procedures for endovascular treatment and antithrombotic agents

If endovascular therapy was indicated, various devices, including coiling systems, intrasaccular devices (e.g., WEB, Contour), intravascular stents, and flow diverters, were used either alone or in combination depending on aneurysm configuration. To reduce periprocedural thromboembolic risk, 3000–5000 IU of heparin (adjusted for patient weight) were administered intraarterial during endovascular procedures.

In procedures using WEB only, all patients received 100 mg of oral acetylsalicylic acid (ASA) as monotherapy daily for six weeks post-intervention. In cases involving stents or flow diverters, a more intensive regimen was applied: an intravenous bolus of 500 mg ASA was administered at the beginning of the procedure, followed by a weight-adjusted continuous infusion of tirofiban over 24 h. After this period, patients were transitioned from tirofiban to prasugrel, with continued administration of ASA (dual therapy) (Ozpeynirci et al., 2019; Rosskopf et al., 2020).

The incidence of in-stent thrombosis in our cohort was <1%, indicating effective PIAT management.

### 2.5 Secondary hemorrhage

The definition of SH included both clinically symptomatic hemorrhages and asymptomatic radiographic findings detected on routine or follow-up imaging. SH were further classified into IH and PH. The latter presented only in gastrointestinal bleeding (GI) and post-catheterization femoral bleeding (PCFB). IH comprised aneurysm rebleeding (AR), access-related hemorrhage (ARH), and PCB. ARH was defined as bleeding occurring within the surgical access area following cranial neurosurgical procedures, such as hemicraniectomy or aneurysm clipping. PCB was defined as new hemorrhage surrounding the catheter trajectory, catheter-associated, and post-EVD intraventricular bleeding. SH was defined broadly to include all intracranial and peripheral bleeding events occurring after the initial aneurysmal rupture. Given the exploratory nature of the study and limited subgroup sizes, detailed risk factor analyses were performed for overall SH, while subtype-specific analyses were descriptive.

### 2.6 Neuroimaging analysis

At least one experienced neurosurgeon evaluated the radiological imaging; additionally, the images were independently reviewed and interpreted by at least one board-certified neuroradiologist.

### 2.7 Cognitive and clinical outcome assessment

Functional and cognitive status were assessed using the mRS and the MoCA, respectively (Wilson et al., 2005; Nasreddine et al., 2005).

The MoCA was performed at the beginning of the initial hospital stay in non-intubated patients, and again 3 months after the event to assess cognitive recovery. Patients who were deceased, not tested within the first two weeks or at FU and those intubated were excluded from MoCA analysis. Patients who had severe deficits preventing test completion were assigned a MoCA score of zero for analytical completeness.

Clinical outcome was assessed using the mRS at discharge and at the 3-month FU. Patients for whom the mRS was not documented at discharge or FU and those intubated were excluded from the respective mRS analyses. Deceased patients received a score of 6.

**Fig. 1:**
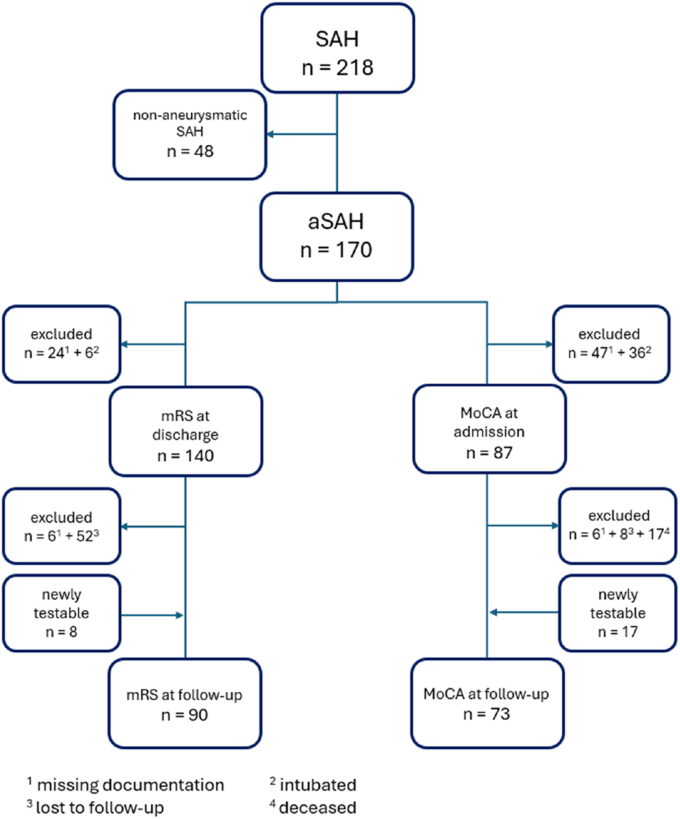
STROBE style flow chart of the cohort included in analysis.

Missing MoCA and mRS data occurred due to loss to FU as well as clinical and organizational factors inherent to routine care, including variability in physician staffing and documentation practices. In some cases, MoCA was not performed or recorded despite eligibility. Furthermore, a subset of patients was not testable due to severe clinical condition (Fig. 1). The proportion of available data at each time point is reported in the Results section.

### 2.8 Data acquisition and statistical evaluation

All data referenced above were retrieved from clinical records, the Picture Archiving and Communication System (PACS), and compiled into an electronic database (Microsoft Excel 2019, Redmond, WA, USA). Data collection was performed by three scientific staff members from the respective departments.

Initial analysis involved descriptive statistical methods. Continuous variables were summarized using means with standard deviations and ranges (minimum/maximum), while categorial variables were described using absolute and relative frequencies. To compare relevant subgroups, independent t-tests were applied for normally distributed quantitative data; for non-normally distributed data, the non-parametric Mann–Whitney *U* test was used. Comparisons of categorical variables were conducted using Fisher's exact test or the chi-squared test, as appropriate. The association between age, as well as WFNS grade, and functional outcome (mRS at discharge and after 3 months) was assessed using Spearman's rank correlation and Kruskal-Wallis testing.

To account for potential confounding, multivariate analyses were conducted for variables that reached a significance level of ≤0.1 in univariate testing as well as clinically relevant variables (age, sex, treatment modality, antiplatelet therapy, ventricular drainage, SV/DCI, and WFNS grade). Specifically, binary logistic regression was employed to identify independent predictors of SH. Multicollinearity was assessed using variance inflation factors (VIF), with all values below 2, indicating no relevant collinearity. Model fit was evaluated using the Hosmer–Lemeshow goodness-of-fit test. Given the non-normal distribution of residuals in linear regression (Shapiro–Wilk test p = 0.024), a sensitivity analysis of dichotomized MoCA scores (≥26 vs. <26) was performed using multivariable logistic regression including secondary hemorrhage, age, vasospasm, treatment modality, and WFNS grade. Functional outcome (mRS at 3 months) was analyzed using ordinal logistic regression including secondary hemorrhage, age, vasospasm, and WFNS grade. The proportional odds assumption was tested using the Brant test. Due to multicollinearity and limited sample size, more complex models including additional procedural variables (e.g., treatment modality and ventricular drainage) were not retained in the final analysis. Analyses were performed on available cases without imputation. Missing data were not replaced due to the retrospective nature of the study.

An exploratory significance level was defined as p ≤ 0.05. Statistical analyses were conducted using the R software for statistical computing (version 4.5.0) and Excel (Microsoft, 2019, Redmond, USA).

## 3 Results

A total of 66 out of 170 patients (39%) developed SH. Demographic and baseline clinical characteristics, as well as initial grading scores, are summarized in Table 1. In univariate analysis, SH occurred more frequently in patients requiring an EVD (91% vs. 65%, p < 0.001) and with higher WFNS grades (IV–V: 53% vs. I–III: 47%, p = 0.034). No significant associations were observed for age, sex, treatment modality, PIAT, i.a. nimodipine, SV/DCI, or Hunt and Hess grades. In multivariable analysis, only EVD was independently associated with the occurrence of SH (OR 5.07, 95% CI 1.9–15.2, p = 0.002). No significant associations were observed for age, sex, treatment modality, PIAT, SV/DCI or severity grade. On the other hand, patients requiring an EVD had significantly higher WFNS grades (p < 0.0001).

**Table 1:** Univariate analysis of patients’ clinical characteristics and initial grading scores.

	SH	No SH	p
**n**	39% (66/170)	61% (104/170)	
**Mean age (SD; min-max)**	58.6 (12.2; 30-84)	56.1 (13.1; 26-85)	0.215
**Male ratio**	26% (17/66)	29% (30/104)	0.661
**Clipping**	30% (20/66)	29% (30/104)	0.839
**Endovascular treatment**	70% (46/66)	71% (74/104)	
**PIAT**	44% (29/66)	47% (49/104)	0.685
**i.a. Nimodipine**	7% (12/66)	6% (11/104)	0.158
**EVD**	91% (60/66)	65% (68/104)	<0.001
**SV/DCI**	23% (15/66)	21% (22/104)	0.809
**Hunt/Hess I-III**	62% (41/66)	73% (76/104)	0.133
**Hunt/Hess IV-V**	38% (25/66)	20% (28/104)	
**WFNS I-III**	47% (31/66)	63% (66/104)	0.034
**WFNS IV-V**	53% (35/66)	37% (38/104)	

### 3.1 Secondary hemorrhage origin

Of the 66 patients with SH, IH occurred in 51 cases (77%). AR accounted for 27% (n = 18), including rebleeding from untreated aneurysms (14%), intra-procedural rupture (3%), and rebleeding despite prior aneurysm occlusion (11%; 2 clipped, 5 endovascularly treated).

ARH was observed in 21% (n = 14), predominantly following craniotomy (15%) or hemicraniectomy (6%), 3 cases required revision surgery.

PCB was frequent (29%, n = 19), with combined PCB and other IH in 9% (1 new cerebellar hemorrhage, 2 AR before treatment, 1 AR after treatment and 2 ARH), requiring revision surgery in 3 cases. No spinal hemorrhage occurred after lumbar drain placement.

**Fig. 2:**
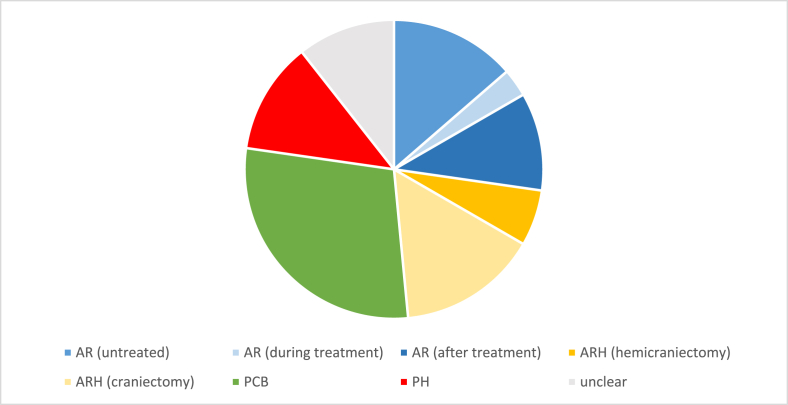
SH in context of origin*.*

PH was uncommon, comprising isolated GI (2%), PCFB (9%), and 1 combined GI and IH event. In 11%, the precise origin of the intracranial hemorrhage remained unclear. This group included one case of new cerebellar hemorrhage, one new bleeding into the internal capsule, one new bleeding into the lateral ventricle, one hemorrhage within vasospasm-related infarct, and three newly developed chronic subdural hematomas in interventionally treated individuals. The distribution of SH origins is summarized in Fig. 2.

A substantial proportion of these events, particularly PCB, were detected on routine imaging and were not necessarily associated with clinical deterioration, whereas 30% (n = 20) showed some form of clinical deterioration leading to or following diagnosis of SH. Of those, 7 showed a decrease in vigilance with 3 receiving revision-surgery and 1 receiving palliative care, 6 had an increase of intracranial pressure with 3 receiving revision-surgery and 2 receiving palliative care, 5 patients developed inguinal hematoma but non required transfusion or surgery. 1 patient had melena and received a transfusion and esophagogastroduodenoscopy with clipping. 1 patient reported self-limiting headache and dizziness. In total, 3 patients with PCB were surgically revised. This data was further limited due to 20% of SH patients (n = 13) being intubated with 8 developing SH before (n = 6) or during (n = 2) aneurysm treatment.

### 3.2 Association with periinterventional antiplatelet therapy

A total of 66 patients developed SH. As shown in Table 2, 26% of these patients were treated with mono-PIAT, 18% with dual-PIAT, and 56% received no PIAT. Across all three groups, IH without associated PH constituted the predominant category of rebleeding events.

**Table 2:** SH in context of PIAT.

	Mono-PIAT	Dual-PIAT	No PIAT
**IH alone**	65% (11/17)	75% (9/12)	84% (31/37)
**-PCB alone**	18% (3/17)	42% (5/12)	30% (11/37)
**-AR alone**	35% (6/17)	17% (2/12)	22% (8/37)
**-ARH alone**	0% (0/17)	0% (0/12)	32% (12/37)
**-other alone**	12% (2/17)	17% (2/12)	0% (0/37)
**PH alone**	6% (1/17)	17% (2/12)	11% (4/37)
**-GI alone**	0% (0/17)	0% (0/12)	3% (1/37)
**-PCFB alone**	6% (1/17)	17% (2/12)	8% (3/37)
**≥2 IH locations**	18% (3/17)	8% (1/12)	5% (2/37)
**IH + PH**	12% (2/17)	0% (0/12)	0% (0/37)

Mono- and dual-PIAT patients exhibited a heterogeneous distribution of hemorrhage types, including isolated PCB, AR and other IH. In contrast, ARH occurred exclusively in patients without PIAT. PCFB were observed across all treatment groups, with a higher percentage in patients that received dual-PIAT. GI was seen exclusively in one patient without PIAT. In total, PH remained relatively uncommon.

Hemorrhages involving two or more intracranial locations were documented in each PIAT group, though most frequently in the mono-PIAT subgroup. Combined intracranial and peripheral bleeding occurred only in mono-PIAT–treated patients. Among the 20 clinically significant SH cases, 13 developed SH prior to initiation of PIAT (with 4 later receiving PIAT), and 7 occurred under ongoing PIAT.

### 3.3 Outcome of the patients after secondary hemorrhage

#### 3.3.1 Modified Rankin Scale

The mRS at discharge was assessed in 140 patients. Missing data were mainly due to incomplete documentation in routine clinical practice (n = 24) and clinical severity (n = 6 intubated patients excluded). Patients with SH showed markedly worse outcomes than those without SH. Only 42% of patients with SH (n = 55) achieved a favorable outcome (mRS ≤3), compared to 69% of patients without SH (n = 85) (p < 0.001). Mortality was higher in the SH group (20% vs. 6%), and severe disability (mRS 5) occurred more frequently (20% vs. 13%).

A subgroup analysis considering both SH and SV/DCI revealed a cumulative detrimental effect. The worst outcomes were observed in patients with both SH and SV/DCI: 42% died (mRS 6) and 42% had moderate/severe disability (mRS 4/5). Patients with SH but no SV/DCI had lower mortality (14%) and a broader distribution across mRS categories. Patients with SV/DCI but no SH demonstrated relatively favorable outcomes, with 37% achieving mRS 0–1 and 16% mortality. The best outcomes were observed in patients with neither complication, with 50% achieving mRS 0-1 and only 3% mortality.

**Fig. 3:**
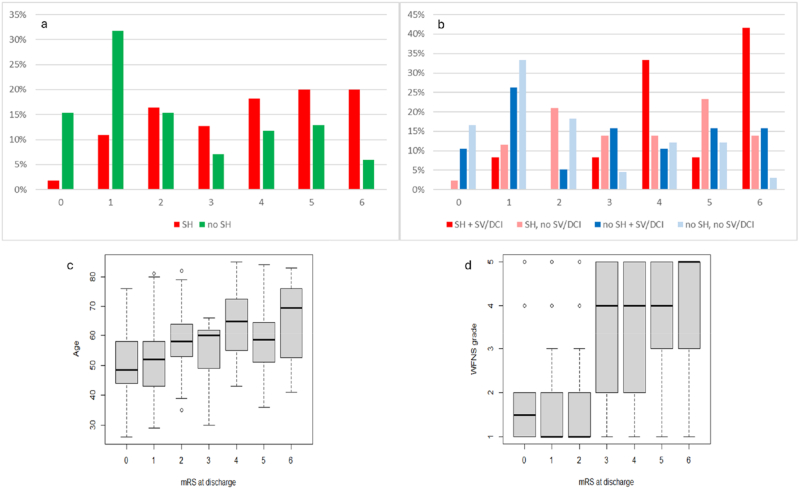
mRS at discharge.

These findings indicate that SH significantly worsens short-term neurological outcomes, an effect that is further amplified when combined with SV/DCI (Fig. 3a and b).

Further subgroup analyses showed that age differed significantly across in-hospital mRS categories (Kruskal–Wallis χ^2^ = 20.9, df = 6, p = 0.002) and showed a significant positive correlation with in-hospital mRS (Spearman's ρ = 0.32, p < 0.001), indicating worse functional outcome with increasing age. A highly significant increasing trend in age across in-hospital mRS categories was observed (Jonckheere–Terpstra p < 0.001). Higher WFNS grades were strongly associated with worse functional outcome as well showing a strong positive correlation with mRS at discharge (Kruskal–Wallis χ^2^ = 36.66, p < 0.001, Spearman's ρ = 0.51, p < 0.001). Ordered trend analyses confirmed a highly significant monotonic increase in mRS with increasing WFNS at discharge (Jonckheere–Terpstra p < 0.01). These results are shown in boxplots Fig. 3c and d.

**Fig. 4:**
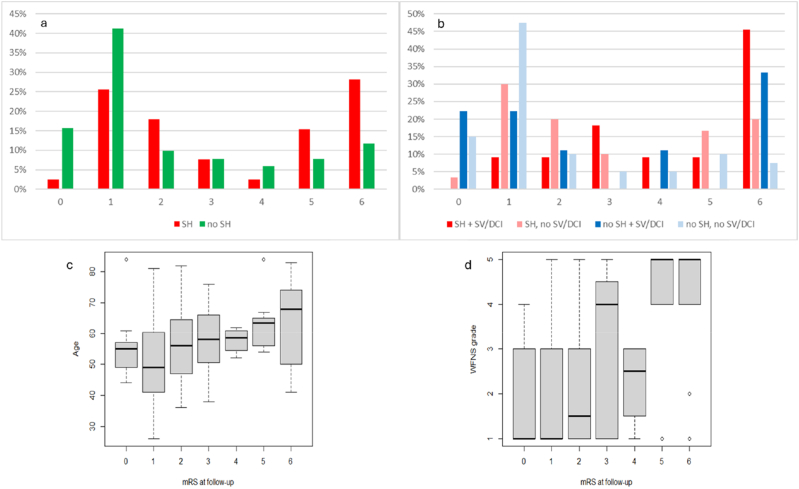
mRS at 3 months FU.

At 3-month FU, mRS data were available for 39 of 66 patients with SH and 51 of 104 patients without SH. Missing follow-up data were mainly attributable to loss to FU as well as incomplete documentation in routine clinical practice. Patients without SH demonstrated significantly better functional outcomes (p < 0.001) (Fig. 4a).

Subgroup analysis by SH and SV/DCI revealed a gradient of outcomes. Patients with both SH and SV/DCI had the worst prognosis, SH patients without SV/DCI showed slightly better recovery. Patients without either complication had the most favorable outcomes (Fig. 4b).

Overall, these results indicate that SH negatively affects functional recovery at 3 months, particularly when occurring alongside SV/DCI, while patients without complications consistently achieve better outcomes.

Further subgroup analyses were made for 3 months FU revealing that age was associated with functional outcome (Kruskal–Wallis χ^2^ = 12.81, p = 0.046). This was supported by a significant monotonic correlation (Spearman's ρ = 0.34, p = 0.001) and a significant trend across mRS categories (Jonckheere–Terpstra p = 0.001). WFNS grade showed an even stronger association with outcome (Kruskal–Wallis χ^2^ = 20.85, p = 0.002), which was confirmed by Spearman correlation (ρ = 0.43, p < 0.001) and Jonckheere–Terpstra testing (p < 0.001). These results are shown in the boxplots Fig. 4c and d.

#### 3.3.2 Montreal Cognitive Assessment (MoCA)

Cognitive function assessed by the MoCA directly after admission before developing SH showed a higher proportion of patients with scores below 26 in the SH group compared to patients without SH (87% vs. 72%, p = 0.015). Missing MoCA data were mainly due to incomplete documentation (n = 47) and clinical severity (n = 36 intubated patients excluded). After 3 months, the proportion of patients with impaired MoCA scores decreased in both groups, indicating significantly more cognitive improvement over time in patients without SH (p < 0.0001) (Table 3).

**Table 3:** MoCA after admission and after 3 months FU.

After admission	SH	No SH	p
<26 points	87% (26/30)	72% (41/57)	0.015
≥26 points	13% (4/30)	28% (16/57)	
**After 3 months**			**p**
<26 points	63% (19/30)	51% (22/43)	<0.0001
≥26 points	36% (11/30)	49% (21/43)	

Analysis of MoCA stratified by the presence of SH and SV/DC demonstrated that patients with both complications exhibited persistent cognitive impairment, with 60% scoring below 26. Among patients with SH but without SV/DCI, 61% scored below 26. In contrast, among patients without SH, those with SV/DCI showed 75% scoring below 26 whereas patients without either complication had the lowest proportion of impaired MoCA scores (46%). Conversely, normal cognitive performance (MoCA ≥26) was most frequently observed in patients without SH or SV/DCI (54%) and was least common in patients with both complications (40%).

Longitudinal analysis of patients with both admission and 3-month MoCA assessments (n = 56) showed that improvement to normal cognition (from <26 to ≥26) occurred in 45% of patients with SH and 28% of patients without SH. Worsening to below 26 was rare (6-10%), and a substantial proportion of patients remained below 26 despite stable or slightly improved scores (45% in SH vs 44% in non-SH). Notably, slight worsening or minor improvement among patients with initially normal MoCA scores was observed only in patients without SH (22%).

Longitudinal cognitive changes were further analyzed according to the origin of SH. Across all SH subtypes, improvement to normal cognition (MoCA ≥26) was observed only in a small number of patients, while worsening to below 26 was rare and occurred sporadically in individual subgroups. The predominant pattern across all hemorrhage origins was no improvement or only minor improvement, with MoCA scores remaining <26 at FU. Notably, no cases of slight worsening or minor improvement were observed among patients with initially normal MoCA scores in any subgroup.

These findings indicate that SH is associated with higher rates of cognitive impairment at admission and persistent deficits at 3 months, particularly when combined with SV/DCI. While some patients improved over time, a significant proportion continued to demonstrate impaired cognitive function despite overall recovery.

#### 3.3.3 Multivariable analyses

In ordinal logistic regression analysis, higher age (OR per year ≈ 1.05, p < 0.001), higher WFNS grade (OR per grade ≈ 1.61, p < 0.001), and vasospasm (OR 2.77, p = 0.043) were independently associated with worse functional outcome (mRS) at FU. In contrast, secondary hemorrhage was not independently associated with outcome (OR 1.78, p = 0.156). The proportional odds assumption was not violated (Brant test p = 0.09). Linear regression was initially performed but violated model assumptions (non-normal residuals). Therefore, a binary logistic regression model of dichotomized MoCA scores (≥26 vs. <26) including secondary hemorrhage, age, vasospasm, treatment modality, and WFNS grade was used. Secondary hemorrhage was not significantly associated with achieving normal cognitive function (OR 0.65, 95% CI 0.21–1.95, p = 0.449). Similarly, age, treatment modality, vasospasm, and WFNS grade were not significantly associated with outcome. Due to missing data because of loss to FU and incomplete documentation, this analysis was limited to a subset of patients (n = 73), and results should therefore be interpreted with caution. The results of the multivariable analyses of mRS and MoCA after 3 months are shown in Table 4, Table 5.

**Table 4:** Results of multivariable analysis regarding mRS after 3 months.

	0	1	2	3	4	5	6	OR	95% CI	p
Age (mean, SD)	56.1 (11.2)	50.9 (11.9)	57 (13.7)	57.9 (11.9)	57.8 (3.8)	63.2 (8.2)	63.9 (13.8)	1.054	1.041 – 1.089	<0.001
SH	1/9	10/31	7/12	3/7	1/4	6/10	11/17	1.783	0.804 – 3.992	0.156
WFNS IV-V	2/9	7/31	2/12	4/7	0/4	8/10	13/17	1.615	1.259 – 2.092	<0.001
SV/DCI	2/9	3/31	2/12	2/7	2/4	1/10	8/17	2.768	1.041 – 7.546	0.043

**Table 5:** Results of multivariable analysis of dichotomized MoCA after 3 months.

	<26 points	≥26 points	OR	95% CI	P
Age (mean, SD)	56.8 (13.4)	52.5 (10.9)	0.979	0.939 – 1.018	0.296
SH	19/41	11/32	0.654	0.212 – 1.954	0.449
Endovascular treatment	36/41	25/32	0.308	0.057 – 1.336	0.133
WFNS IV-V	15/41	6/32	0.507	0.144 – 1.669	0.270
SV/DCI	9/41	4/32	0.691	0.162 – 2.610	0.594

## 4 Discussion

### 4.1 Origin of rebleeding

The definition of SH after aSAH comprised a heterogeneous group of events with different underlying mechanisms and clinical relevance. Importantly, the reported incidence of SH (39%) included both clinically significant hemorrhages and asymptomatic radiographic findings, particularly PCB detected on routine imaging. While this comprehensive approach allows a broad assessment of bleeding complications, it limits the ability to draw conclusions for specific subtypes, may limit comparability with previous studies focusing on clinically relevant bleeding events only and may partly explain the relatively high observed incidence. In our cohort, IH accounted for the majority (77 %) of SH events. Among these, PCB represented the most frequent subtype (29%), followed by AR (27 %). This distribution highlights that non-aneurysmal mechanisms contribute substantially to the overall burden of SH. However, given the limited sample size, subgroup analyses were restricted to descriptive reporting and should be interpreted cautiously.

AR remains a clinically critical entity. In our cohort, rebleeding after aneurysm treatment occurred in a small proportion of patients (4%), consistent with previously reported low rates early after treatment (Willinsky et al., 2009), while long-term randomized data confirm that recurrent hemorrhage from treated aneurysms, although rare, may still occur (Molyneux et al., 2005). These events likely reflect factors such as aneurysm size (Boogaarts et al., 2015), incomplete aneurysm exclusion (Pierot et al., 2010; Johnston et al., 2008), timing of intervention (Kienzler et al., 2016) and EVD (Cagnazzo et al., 2020), procedural complications, or persistent vascular fragility. In addition, ruptures of untreated aneurysms (5%) and intra-procedural hemorrhages (1%) contributed to the overall incidence of SH, underlining the multifactorial nature of hemorrhagic risk in the acute phase (Molyneux et al., 2005; Johnston et al., 2008).

PCB emerged as a frequent contributor to and the only variable independently associated with SH (OR 5.07, 95% CI 1.9–15.2, p = 0.002) in our cohort, accounting for 37% of IH. However, this finding must be interpreted with caution, as patients requiring EVD typically present with more severe hemorrhage and worse clinical status, introducing potential confounding by indication. Previous studies have shown that imaging-detected hemorrhage along ventricular catheter tracts is relatively common, with frequencies ranging from ∼5% up to ∼20%, whereas clinically significant bleeding remains rare (Sawarkar et al., 2024; Ko et al., 2014; Dey et al., 2012). These findings suggest that PCB represents a predominantly radiographic phenomenon in many cases, which should be considered when interpreting overall SH incidence.

ARH associated with surgical procedures also contributed to SH (21%). These events reflect procedure-related tissue injury and differ mechanistically from aneurysm-related bleeding. Although often clinically less severe, they may complicate postoperative management and contribute to overall morbidity (Kalfas and Little, 1988; Budohoski et al., 2023).

PH was less common but remains a component of the SH spectrum, illustrating that secondary bleeding after aSAH is not limited to aneurysm- or access-related events. Finally, in 11 % of cases, the precise bleeding origin could not be determined, reflecting the complexity of post-aneurysmal SAH hemorrhagic pathology.

Taken together, these findings illustrate that SH after aSAH arises from multiple mechanisms, including aneurysm-related, procedural, and other causes. The relative contribution of each subtype in our cohort should be interpreted in the context of the broad SH definition and limited subgroup sizes. The high incidence of PCB and ARH, together with AR, highlights the need for meticulous attention to procedural technique and postoperative care. Future studies should aim to integrate morphological risk factors, procedural timing, and individualized bleeding profiles to optimize prevention strategies and reduce the morbidity and mortality associated with SH.

### 4.2 The correlation of periinterventional antiplatelet therapy and secondary hemorrhagic complications

This study further explored the association between PIAT and SH in patients with aSAH. While previous studies showed that antiplatelet therapy may increase bleeding risk by impairing platelet aggregation, particularly in the context of invasive procedures such as EVD placement (Merritt and Bhatt, 2002; Carpenter et al., 2016; Li et al., 2023), no statistically significant association between PIAT and SH was observed in our cohort (all p > 0.3). Importantly, this finding must be interpreted with caution, as the present study is likely underpowered to detect clinically meaningful differences in hemorrhagic risk related to antiplatelet therapy.

Descriptive subgroup analyses suggested differences in the distribution of hemorrhage types across PIAT groups. However, as no formal statistical comparisons were performed because of limited subgroup size, these observations should be considered exploratory and hypothesis-generating only. Notably, patients receiving dual antiplatelet therapy showed numerically lower rates of SH compared to those receiving monotherapy. This counterintuitive finding most likely reflects selection bias and small subgroup sizes rather than a true protective effect.

A key limitation of the present study is the lack of systematic documentation of the temporal relationship between EVD placement and initiation of PIAT. This limits the ability to assess potential interactions between procedural and pharmacological risk factors, which have been suggested to play a critical role in hemorrhagic complications (Kung et al., 2011; Bruder et al., 2015). Previous studies have reported conflicting results regarding the impact of antiplatelet therapy on hemorrhagic complications. While some data suggest an increased risk of procedure-related bleeding (Olexa et al., 2024; Rowe et al., 2018), others have not demonstrated a significant association and instead highlight the role of other factors such as individual coagulopathy, catheter trajectory, or device design (Qin et al., 2021; Tang et al., 2024). Differences in study design, patient selection, and treatment protocols may account for these inconsistencies. Overall, our findings do not support a significant association between PIAT and SH. However, given the retrospective design, limited sample size, lack of temporal resolution, and heterogeneity of bleeding definitions, causal conclusions cannot be drawn. Taken together, these results should be considered hypothesis-generating. Prospective, ideally multicenter studies with standardized documentation of treatment timing are required to better define the relationship between PIAT, EVD placement, and SH risk (Darkwah Oppong et al., 2020; Lee et al., 2023).

### 4.3 Neurological and cognitive outcomes: modified Rankin Scale and Montreal Cognitive assessment

SH was associated with significantly worse short-term neurological outcomes in our cohort. At discharge, patients with SH achieved functional independence (mRS ≤3) substantially less often than patients without SH, a difference that persisted at the 3-month FU: These findings highlight the clinically relevant impact of SH on early functional recovery after aSAH, though results may be influenced by the substantial proportion of missing follow-up data.

Cognitive assessment using the MoCA revealed lower scores at admission in patients who later developed secondary hemorrhage, identifying a clinically more vulnerable subgroup. However, as baseline MoCA was performed prior to the occurrence of SH, these differences most likely reflect greater initial disease severity rather than an effect of SH. At 3-month FU, a higher proportion of patients with SH continued to demonstrate impaired cognitive performance (MoCA <26) compared to those without SH. However, multivariable analysis did not identify SH as an independent predictor of cognitive outcome. Longitudinal analyses further showed that a proportion of patients with SH improved from impaired to normal cognitive performance over time. These findings suggest that, while cognitive impairment remains common after SH, recovery trajectories are heterogeneous and may not be solely attributable to SH.

Multivariate analyses identified age and initial WFNS grade as the most robust predictors of functional outcome, whereas SH did not emerge as an independent determinant of long-term recovery. In contrast to functional outcome, no independent predictors of cognitive outcome could be identified in multivariable analysis. This may reflect the multifactorial nature of cognitive recovery after aSAH, as well as limited statistical power due to sample size and missing data. These findings suggest that the observed differences in cognitive outcomes may be primarily driven by baseline disease severity rather than secondary hemorrhage itself. More complex models including additional covariates were not stable, highlighting the limitations imposed by sample size and inter-variable correlations. Our findings are consistent with previous studies identifying age and initial disease severity as the strongest predictors of outcome after aSAH (Macdonald, 2013; Al-Khindi et al., 2010). This pattern suggests that SH reflects a marker of overall disease severity and clinical complexity rather than an independent prognostic determinant of long-term functional or cognitive outcome.

In addition, SV/DCI was independently associated with worse functional outcome in multivariable analysis, underscoring its role as a key contributor to secondary brain injury after aSAH. This finding aligns with existing literature and highlights the importance of both early brain injury and delayed complications in determining patient outcome.

Overall functional recovery in this cohort was moderate despite the high burden of complications, which may in part reflect timely aneurysm securing, standardized neurocritical care, and treatment in a specialized high-volume center, factors previously associated with improved outcomes after SAH (Nobels-Janssen et al., 2024).

Importantly, discrepancies between functional and cognitive trajectories were observed. While patients with SH continued to show worse functional outcomes at follow-up, cognitive recovery appeared more heterogeneous. A substantial proportion of patients, including those with SH, demonstrated improvement over time, although many remained below the threshold for normal cognitive function. These findings underscore that global disability scales such as the mRS may not fully capture persistent cognitive deficits. Consistent with prior reports, patients with favorable functional outcomes may still exhibit clinically relevant cognitive impairment, particularly in executive and memory domains (Geraghty et al., 2020; Wong et al., 2014). These findings emphasize the importance of integrating both functional and cognitive assessments to comprehensively evaluate recovery after aneurysmal SAH.

## 5 Limitations

Several limitations of this study should be considered. First, the definition of SH included both clinically significant and asymptomatic radiographic events, particularly PCB detected on routine imaging. This broad definition introduces heterogeneity, may overestimate the clinical impact of SH, and limits comparability with studies focusing exclusively on clinically relevant hemorrhages. Second, the retrospective design introduces an inherent risk of bias, including confounding by indication. Patients requiring EVD placement are likely to differ systematically in disease severity and treatment complexity, which limits causal interpretation of the observed associations. Third, the study was not powered to detect differences in hemorrhagic risk related to antiplatelet therapy, particularly in subgroup analyses such as mono-versus dual-PIAT. The counterintuitive finding of numerically lower bleeding rates in patients receiving dual antiplatelet therapy most likely reflects selection bias and small subgroup sizes rather than a true protective effect. In addition, the exact temporal relationship between EVD placement, aneurysm treatment, and initiation of PIAT was not consistently documented, precluding a more detailed analysis of potential interactions between procedural and pharmacological risk factors. Fourth, although multivariable analysis identified an association between SH and EVD placement, this finding must be interpreted with caution, as the high prevalence of EVD use in patients with SH likely reflects greater initial disease severity rather than an independent causal effect. Fifth, missing outcome data, particularly for 3-month mRS and MoCA, may introduce attrition bias and limit the robustness of long-term outcome analyses. Outcome data were incomplete due to clinical constraints, further reducing statistical power and potentially introducing selection bias.

Sixth, SH diagnosis was based on imaging and clinical assessment without centralized or blinded adjudication, which may introduce observer bias. Finally, long-term cognitive outcomes beyond three months were not assessed, limiting conclusions on the sustained impact of SH on cognitive recovery. Sex-specific analyses were not performed due to the limited sample size, which may further restrict generalizability.

Future studies should distinguish between clinically relevant and radiographic-only hemorrhages to allow more precise risk stratification. Prospective, ideally multicenter studies with standardized documentation of treatment timing are required to better define the relationship between PIAT, EVD placement, and SH risk.

## 6 Conclusion

SH following aSAH occurred in 39% of patients in our cohort and was associated with worse early neurological and cognitive outcomes. Intracranial SH predominated, with PCB and AR representing the most frequent subtypes, while ARH further contributed to the overall burden. EVD placement emerged as the strongest factor associated with SH. However, this finding should be interpreted with caution, as it likely reflects underlying disease severity and treatment complexity rather than a purely causal relationship. These observations nevertheless highlight the importance of meticulous procedural technique, careful catheter placement, and consideration of image guidance in selected cases. No statistically significant association between PIAT and SH was observed. Given the limited sample size, lack of standardized documentation of treatment timing, and potential confounding by indication, the study was underpowered to reliably assess the impact of antiplatelet therapy or differences between mono- and dual-PIAT. The interaction between pharmacological and procedural risk factors therefore remains insufficiently understood. Importantly, age and initial WFNS grade were confirmed as robust predictors of functional outcome, whereas SH did not independently predict long-term outcome in multivariable analysis, suggesting that SH may primarily represent a marker of disease severity rather than an independent determinant of recovery. Overall, these findings underscore the multifactorial nature of hemorrhagic complications after aSAH. While causal relationships cannot be definitively established, individualized risk assessment, optimized procedural strategies, and careful periinterventional management remain central to minimizing complications. Prospective, adequately powered studies with standardized documentation of treatment timing are needed to better define the interplay between EVD placement, antiplatelet therapy, and SH risk.

## Data availability statement

The authors will make the raw data underlying the conclusions of this article available upon request, without unnecessary limitations.

## Ethics statement

The study was reviewed and approved by the Ethics Committee of the University of Ulm (Approval No. 280/21) and conducted in accordance with the principles outlined in the Declaration of Helsinki. Informed consent for the use of anonymized medical data for research purposes was obtained from all patients or their legally authorized representatives.

## Author contributions

VM: conception, data interpretation and manuscript writing. VM, MA, AZ: data collection. VM, LM, BM: statistics and interpretation. GD, AP: study conception, editing and review. DW, AZ, MA, RK, TK, BM, JR, LM, CRW: Editing and review. All authors contributed to the article and approved the submitted version.

## Funding statement

This research did not receive any specific grant from funding agencies in the public, commercial, or not-for-profit sectors. The study was conducted as part of the authors’ employment at the University of Ulm, Department of Neurosurgery. The employer was not involved in the writing, editing, approval, or decision to publish the manuscript.

## Declaration of competing interest

The authors declare that they have no known competing financial interests or personal relationships that could have appeared to influence the work reported in this paper.
